# Relationship between DNA mismatch repair and CRISPR/Cas9-mediated knock-in in the bovine **β**-casein gene locus

**DOI:** 10.5713/ab.21.0117

**Published:** 2021-06-24

**Authors:** Seung-Yeon Kim, Ga-Yeon Kim, Hyeong-Ju You, Man-Jong Kang

**Affiliations:** 1 Department of Animal Science, College of Agriculture and Life Science, Chonnam National University, Gwangju 61186, Korea

**Keywords:** Cadmium Chloride (CdCl_2_), Clustered Regularly Interspaced Short Palindromic Repeats/CRISPR-associated 9 (CRISPR/Cas9), Homologous Recombination (HR), Knock-in, Mismatch Repair (MMR), RAD51 Recombinase (RAD51)

## Abstract

**Objective:**

Efficient gene editing technology is critical for successful knock-in in domestic animals. RAD51 recombinase (*RAD51*) gene plays an important role in strand invasion during homologous recombination (HR) in mammals, and is regulated by checkpoint kinase 1 (*CHK1*) and *CHK2* genes, which are upstream elements of RAD51 recombinase (RAD51). In addition, mismatch repair (MMR) system is inextricably linked to HR-related pathways and regulates HR via heteroduplex rejection. Thus, the aim of this study was to investigate whether clustered regularly interspaced short palindromic repeats/CRISPR-associated 9 (CRISPR/Cas9)-mediated knock-in efficiency of human lactoferrin (hLF) knock-in vector in the bovine β-casein gene locus can be increased by suppressing DNA MMR-related genes (*MSH2*, *MSH3*, *MSH6*, *MLH1*, and *PMS2*) and overexpressing DNA double-strand break (DSB) repair-related genes (*RAD51*, *CHK1*, *CHK2*).

**Methods:**

Bovine mammary epithelial (MAC-T) cells were transfected with a knock-in vector, RAD51, CHK1, or CHK2 overexpression vector and CRISPR/sgRNA expression vector to target the bovine β-casein gene locus, followed by treatment of the cells with CdCl_2_ for 24 hours. After 3 days of CdCl_2_ treatment, the knock-in efficiency was confirmed by polymerase chain reaction (PCR). The mRNA expression levels of DNA MMR-related and DNA DSB repair-related genes were assessed by quantitative real-time PCR (RT-qPCR).

**Results:**

Treatment with CdCl_2_ decreased the mRNA expression of RAD51 and MMR-related genes but did not increase the knock-in efficiency in MAC-T cells. Also, the overexpression of DNA DSB repair-related genes in MAC-T cells did not significantly affect the mRNA expression of MMR-related genes and failed to increase the knock-in efficiency.

**Conclusion:**

Treatment with CdCl_2_ inhibited the mRNA levels of RAD51 and DNA MMR-related genes in MAC-T cells. However, the function of MMR pathway in relation to HR may differ in various cell types or species.

## INTRODUCTION

The efficiency of gene targeting is still low due to the limited opportunity for spontaneous gene targeting in somatic cells and random integration of exogenous genes into the genome [1]. Genome editing with engineered nucleases, such as zinc finger nucleases (ZFNs), Transcription activator-like effector nucleases (TALENs), and clustered regularly interspaced short palindromic repeats/CRISPR-associated 9 (CRISPR/Cas9) system, is a powerful technique that facilitates substantially higher levels of accurate genome editing at endogenous gene loci than traditional methods [2,3].

As noted above, these technologies can introduce DNA double-strand breaks (DSBs) in a specific gene locus and the cells repair DNA damage via several DNA repair pathways, including non-homologous end joining (NHEJ) and homologous recombination (HR) [4]. The NHEJ pathway usually induces indels in DNA sequences that may result in functional loss in the gene [5]. In contrast, the HR pathway is homology-dependent that resects DSB ends and synthesizes DNA using homologous DNA templates [6].

However, despite the availability of ZFNs, TALENs or CRISPR/Cas9 system, the efficiency of gene targeting by HR was reported to be in the range of 0.5% to 20% [7–9] since the frequency of gene targeting by HR is 1,000-fold lower than NHEJ [10]. Hence, to facilitate knock-in of exogenous gene into the genome, many studies designed to enhance HR efficiency have been carried out recently [11,12].

It is widely accepted that RAD51 recombinase (RAD51) is an important player in HR and the repair of DNA DSBs [13]. RAD51 can be regulated by checkpoint kinase 1 (CHK1) or CHK2 [14]. CHK1 and CHK2 are basically involved in cell cycle, and also play a role in RAD51 recruitment to the sites of DNA damage [15].

Recent studies have reported that HR is inextricably linked with mismatch repair (MMR) [16]. DNA MMR is a highly conserved post-replication repair system that recognizes base-base replacement, indels and other DNA errors [17]. Homologous and homeologous recombination rates were increased in MMR-defective strains [18]. It is also reported that down-regulation of MutS complex, which are involved in initiation of MMR, can be generated by cadmium-induced oxidative stress [19]. Therefore, it is necessary to suppress the MMR mechanism to increase HR-mediated knock-in efficiency.

However, in spite of numerous studies investigating cadmium, MMR, and DNA DSB repair-related genes, the correlation between MMR mechanism and knock-in efficiency has yet to be determined in bovine cells. Accordingly, the aim of this study was to investigate the correlation between MMR and HR system in mammalian cells and to develop efficient gene editing tools mediated via CRISPR/Cas9. In this study, in an attempt to increase the knock-in efficiency of human lactoferrin (*hLF*) gene in the β-casein locus of bovine mammary epithelial (MAC-T) cells, the expression levels of DNA MMR-related genes were inhibited by cadmium chloride (CdCl_2_) treatment, and DNA DSB repair-related genes (*RAD51*, *CHK1*, or *CHK2*) were overexpressed.

## MATERIALS AND METHODS

### Cell culture and transfection

The MAC-T cell were cultured in the growth medium: Dulbecco’s modified Eagle medium (Hyclone, Logan, UT, USA) supplemented with 10% fetal bovine serum (Hyclone, USA), 1% penicillin/streptomycin (Hyclone, USA) and 0.05% gentamicin (Sigma-Aldrich Inc., St. Louis, MO, USA). MAC-T cells were seeded at a density of 1.75×10^5^ cells per well into 12-well plates (SPL, Gyeonggi, Pocheon, Korea) or 3.0×10^5^ cells per well into 6-well plates (SPL, Korea) and incubated at 37°C with 5% CO_2_. The culture medium was replaced with 0.5 or 1 mL of fresh media before transfection. Thereafter, the cells were co-transfected with circular hLF knock-in vector II (1.25 or 2 μg), CRISPR-sgRNA expression vector (0.625 or 1 μg) and overexpression vectors of DNA DSB repair-related genes (*bRAD51*, *bCHK1*, or *bCHK2*) (0.625 or 1 μg) using Xfect transfection reagent (Takara, Tokyo, Japan) according to the manufacturer’s instructions. Totally, 2.5 or 5 μg of DNA was added to each well and the total volume was made up to 50 or 100 μL with Xfect reaction buffer, followed by the addition of 0.75 or 1.5 μL of Xfect polymer. After 24 hours of transfection, the treatment groups were treated with 1, 10, or 25 μM CdCl_2_ and the medium was replaced with fresh medium the next day. Cells were incubated for further 48 hours, followed by the extraction of genome DNA from the transfected cells using the G-DEX IIc genomic DNA extraction kit (for cell/tissue) (Intron, Seongnam-si, Korea) according to the manufacturer’s instructions.

### Construction of the knock-in vector

To construct the knock-in vector II, we first modified the 3′ homology arm of pBSK(−)m_RG1_1 l kb 5′ arm_hLF_BGHpolyA_EGFP_BGHpolyA _2 kb 3′ arm, termed hLF knock-in vector I, which contains approximately 2 kb homology arm at 3′ site and was previously reported by Kim et al [20]. The 3′ arm used for knock-in vector was amplified by polymerase chain reaction (PCR) from genome DNA of MAC-T cells. The 1 kb 3′ arm fragment is homologous to a region of exon 3 in the β-casein gene and starts exactly from the Cas9/sgRNA cleavage site, which was previously reported by Jeong et al [21]. PCR was performed using the bβCE3 II S primer (AAGCTTACCTGGTGAGGTAAGATATTTTTAT) and the bβCE3 1kbPHR II AS primer (GTCGACCAGAT TTAGGACTACACTCA), containing HindIII and SalI restriction enzyme sites using KOD FX Neo (Toyobo, Osaka, Japan). The purified PCR fragment was sub-cloned into pGEM-T easy vector (Promega, Madison, WI, USA) and the primary structure of the fragment was confirmed via sequencing using T7 and SP6 primers. Finally, to construct the knock-in vector II, the pGEM T-easy_β-casein exon 3 3′ arm plasmid was digested by Hind III and Sal I enzymes to obtain the bβCE3 3′ arm. The fragment was then inserted into the Hind III-Sal I site of pBSK(−)m_RG1_1 kb 5′ arm_hLF_BGHpolyA_EGFP_BGHpolyA_2 kb 3′ arm plasmid to produce pBSK(−)m_RG1_1 kb 5′ arm_hLF_BGHpolyA_EGFP_BGHpolyA_1 kb 3′ arm plasmid, designated as hLF knock-in vector II.

### Analysis of knock-in efficiency by polymerase chain reaction

Knock-in efficiency of hLF knock-in vector in MAC-T cells was confirmed by first and second PCRs. The genome DNA from each group was used as a DNA template for PCR. The first PCR was performed with RG1_hLF 5sc S1 primer (GC TCCTCCTTCACTTCTTGTCCTCTACTTT) and hLFsc AS1 primer (AACTCACTATTATGCCGTGGCTGTGGT GAA), harboring outside of 5′ arm and *hLF* gene each in hLF knock-in vector II using KOD FX Neo (Toyobo, Japan). The PCR entailed denaturation at 95°C for 15 minutes, followed by 25 cycles of amplification at 95°C for 20 seconds, annealing at 66°C for 40 seconds, strand extension at 72°C for 2.5 minutes, and a final elongation step at 72°C for 5 minutes. Then, the second PCR was carried out using the RG1_hLF 5sc S4 primer (CAAATTACGAATTAGCATC CATGAGAAAAA) and the hLFsc AS4 primer (CTTGTC TTCCTCG TCCTGCTGTTCCTCGGG) using KOD FX Neo (Toyobo, Japan) as follows: denaturation at 95°C for 15 minutes, followed by 30 cycles of amplification at 95°C for 20 seconds, annealing at 58°C for 40 seconds, strand extension at 72°C for 1.5 minutes, and a final elongation step at 72°C for 5 minutes. The PCR fragments were confirmed by electrophoresis on a 0.8% agarose gel. For comparative quantification, each DNA band was normalized to the bβCE7 region.

### Quantitative real-time polymerase chain reaction analysis

Total RNA from MAC-T cells was isolated using RNeasy mini kit (QIAGEN, Hilden, Germany). Next, for cDNA synthesis, 2 μg of total RNA was used as a template and RT-PCR was performed using random primers and Superscript II reverse transcriptase (Invitrogen, Carlsbad, CA, USA) according to the manufacturer’s protocol. Quantitative real-time PCR (RT-qPCR) was carried out to measure the mRNA expression of RAD51 and DNA MMR-related genes in a total volume of 20 μL, containing 20 ng cDNA, 10 pmol of forward and reverse primer pairs (Table 1), and 10 μL of TOPreal qPCR 2X PreMIX (Enzynomics, Daejeon, Korea). All reactions were repeated three times and were run by Mx3000P (Agilent Technologies, Santa Clara, CA, USA) under the following conditions: denaturation at 95°C for 10 minutes, followed by 40 cycles of amplification at 95°C for 10 seconds, annealing at 60°C for 15 seconds, and strand extension at 72°C for 15 seconds. For comparative quantification, mRNA expression levels of each gene were normalized to the mouse Rplp0 transcript.

### Western blot analysis

Protein from MAC-T cells was extracted using PRO-PREP protein extraction solution (Intron, Korea) according to the manufacturer’s instructions. Protein extracts (50 μg) derived from the cells were resolved by 8% or 12% Sodium dodecyl sulfate-polyacrylamide gel electrophoresis (SDS-PAGE) at 60 mA for 1.5 hours and electroblotted onto polyvinylidene fluoride membranes for protein blotting (Bio-Rad Co., Hercules, CA, USA) at 80 V for 2 hours in a trans-blot apparatus (Bio-Rad Co., USA) using Tris-glycine buffer. After blocking the membrane for 1 hour at room temperature in 5% skim milk in Tris-buffered saline with 0.1% Tween 20 (TBST), the blot was probed with rabbit polyclonal anti-*MSH2* antibody (Abcam, Cambridge, UK) at a dilution of 1:5,000 or mouse monoclonal anti-*RAD51* antibody (Santa Cruz Biotechnology, Dallas, TX, USA) at a dilution of 1:1,000 in 5% skim milk in TBST overnight at 4°C. After overnight incubation, the membrane was washed with TBST three times and incubated with horseradish peroxidase (HRP)-conjugated goat anti-rabbit immunoglobulin G (IgG) or HRP-conjugated goat anti-mouse IgG at a dilution of 1:1,000 in 5% skim milk in TBST at room temperature for 2 hours. The membrane was washed three times with TBST, and *MSH2* and *RAD51* proteins were detected using EZ-Western Lumi Pico Kit and EZ-Western Lumi Femto Kit (Dogen, Seoul, Korea). To remove bound antibody and facilitate the reuse of the blot, the membrane was incubated in SDS/β-mercaptoethanol solution at 55°C for 20 minutes. The antibody was stripped off, and the membrane was washed three times with TBST, followed by steps as described in the western blot protocol above. The blot was re-probed with mouse monoclonal β-actin antibody (Santa Cruz Biotechnology, USA) at a dilution of 1:1,000 in 5% skim milk in TBST, following blocking at 4°C overnight. The membrane was then washed with TBST three times and incubated with HRP-conjugated goat anti-mouse IgG at a dilution of 1:2,000 in 5% skim milk in TBST at room temperature for 2 hours. After membrane was washed three times with TBST, β-actin protein was detected using EZ-Western Lumi Pico Kit (Dogen, Korea). For comparative quantification of protein levels, *RAD51* and *MSH2* protein bands were normalized to β-actin, respectively.

### Statistical analysis

Densitometric quantification of DNA or protein bands was performed by analyzing each data using UN-SCAN-IT gel Analysis Software (Silk Scientific Inc., Orem, UT, USA). Subsequently, a statistical analysis of the mRNA levels and densitometric quantification of DNA and protein bands was performed with GraphPad Prism 5 (GraphPad Software Inc., San Diego, CA, USA). The data were analyzed in either of the two ways. First, the data were analyzed with one-way analysis of variance, followed by Dunnett test. This method was used to compare all columns with reference to the control column. Second, a t-test was used for comparison of differences between the two columns. The confidence interval was set to 95%.

## RESULTS

### Construction of the knock-in vector expressing *hLF* gene in the bovine **β**-casein gene locus

**Figure 1:**
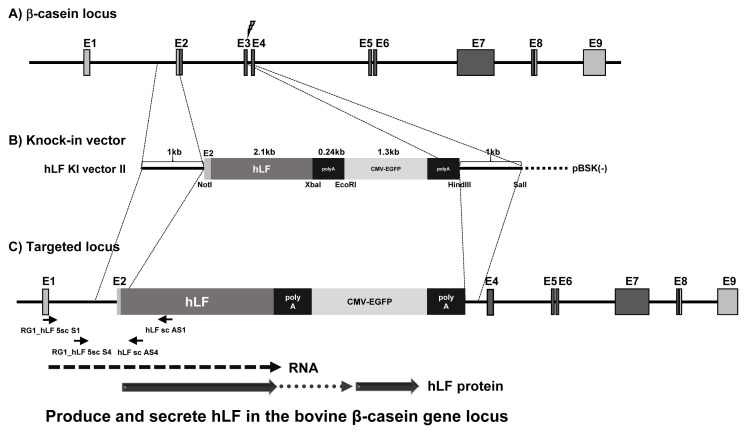
The knock-in strategy targeting the bovine β-casein gene locus. The diagram shows the targeting strategy of knock-in vectors by HR in the β-casein gene locus. (A) Genome structure of the β-casein gene locus. (B) hLF knock-in vector II. (C) Targeted structure of knock-in vector by HR. The hLF transgene is expressed by β-casein gene regulatory DNA sequence and mRNA is translated with β-casein by ribosome. The hLF protein is synthesized and secreted using the β-casein gene locus. The polymerase chain reaction primer pairs used for detecting HR events are indicated by arrows. HR, homologous recombination; hLF, human lactoferrin.

The knock-in vector was constructed for expression of the bovine β-casein fused *hLF* gene in the bovine mammary gland as shown in the schematic diagram (Figure 1). The knock-in vector is composed of a 5′ homology arm, *hLF* gene, BGH poly A signal, cytomegalovirus-enhanced green flurescent protein (*CMV-EGFP*) gene and 3′ homology arm. Poly A signal was attached to stabilize the expression of foreign genes. *EGFP* gene was used as a positive selection marker to visualize the expression of the knock-in vector in the cells. The knock-in vector was designed to carry a 3′ arm that was homologous to an exon 3 region in the bovine β-casein gene locus and starting exactly at the cleavage site by CRISPR-sgRNA system targeting β-casein exon 3. If the knock-in vector undergoes HR in the bovine β-casein gene locus, hLF can be expressed under the control of β-casein promoter and gene regulatory sequences.

### CRISPR/Cas9-mediated knock-in efficiency and expression levels of RAD51 and DNA MMR-related genes in MAC-T cells treated with CdCl**_2_**

**Figure 2:**
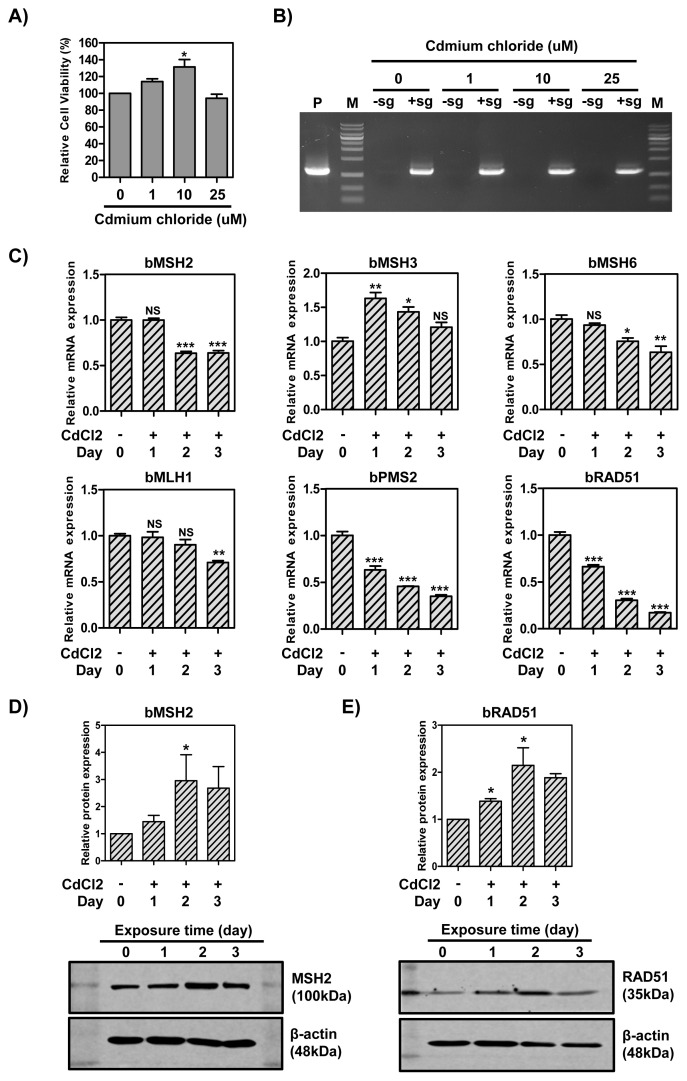
Knock-in efficiency and expression levels of RAD51 and DNA MMR-related genes in MAC-T cells treated with CdCl_2_. (A) Cell viability. (B) Polymerase chain reaction analysis of knock-in efficiency. PC, positive control; M, DNA size marker (1 kb ladder). (C) The mRNA levels of Mut S genes (*MSH2*, *MSH3*, and *MSH6*), Mut L genes (*MLH1* and *PMS2*) and *RAD5*1 gene. (D)-(E) Protein expression of MSH2 and RAD51. RAD51, RAD51 recombinase; MMR, mismatch repair. Error bars show the standard deviation from each sample. NS, no statistical difference; * p<0.05; ** p<0.01; *** p<0.0001.

To determine whether CdCl_2_ treatment affected cell viability in MAC-T cells, we treated the cells with different concentrations of CdCl_2_. In response to CdCl_2_ treatment, MAC-T cell viability ratios were increased at 1 and 10 μM (114.05% and 131.38% each) and decreased at 25 μM (94.08%) compared with the untreated control (Figure 2A). No viable cells were found at 50 μM (data not shown). Statistical analysis did not reveal any notable differences between the groups. Next, the impact of CdCl_2_ concentration on knock-in efficiency in MAC-T cells was analyzed by transfecting the cells with hLF knock-in vector II and CRISPR-sgRNA expression vector, followed by treatment with CdCl_2_ (0 to 25 μM). As a result, the knock-in efficiency in the presence of CRISPR-sgRNA expression vector was significantly higher than in the absence of the expression vector. However, no detectable change in knock-in efficiency was observed between the CdCl_2_ treatment groups (Figure 2B). Based on these results, the cells were treated with 1 μM CdCl_2_ in subsequent experiments to minimize cell death and mutation rate.

Next, to determine whether the treatment times of CdCl_2_ affected the mRNA levels of RAD51 and DNA MMR-related genes, MAC-T cells were treated with 1 μM CdCl_2_ for 3 days and the total RNA was extracted daily. As indicated in Figure 2C, our experiments detected decreased levels of mRNA expression in MutSα (MSH2 and MSH6) and MutLα (MLH1 and PMS2) homologs, and RAD51 by increasing the duration of CdCl_2_ treatment. Compared with the control, the mRNA levels of MSH2 and MSH6 on day 3 were decreased by 36%, MLH1 was decreased by 29%, and PMS2 was decreased by 66%. In contrast, MSH3 mRNA levels were increased on day 1 and decreased on day 2 and 3, but were slightly higher than the control levels (1.2-fold).

To determine the influence of different treatment times of CdCl_2_ on the expression of MSH2 and RAD51 proteins, a western blot was carried out and normalized to β-actin. Compared with the control, the levels of MSH2 protein were increased on day 1 and 2 (1.2-fold and 3.3-fold, respectively) and decreased on day 3 (Figure 2D). Moreover, the expression of RAD51 protein was increased on day 1 and 2 (1.22-fold and 2.32-fold, respectively) compared with the control, and decreased on day 3 (Figure 2E). However, no significant difference was observed between day 0 and 3. Consequently, CdCl_2_ treatment decreased the mRNA levels of DNA MMR-related genes but did not decrease the expression of MSH2 protein.

### CRISPR/Cas9-mediated knock-in efficiency and expression levels of DNA MMR-related genes in MAC-T cells overexpressing RAD51

**Figure 3:**
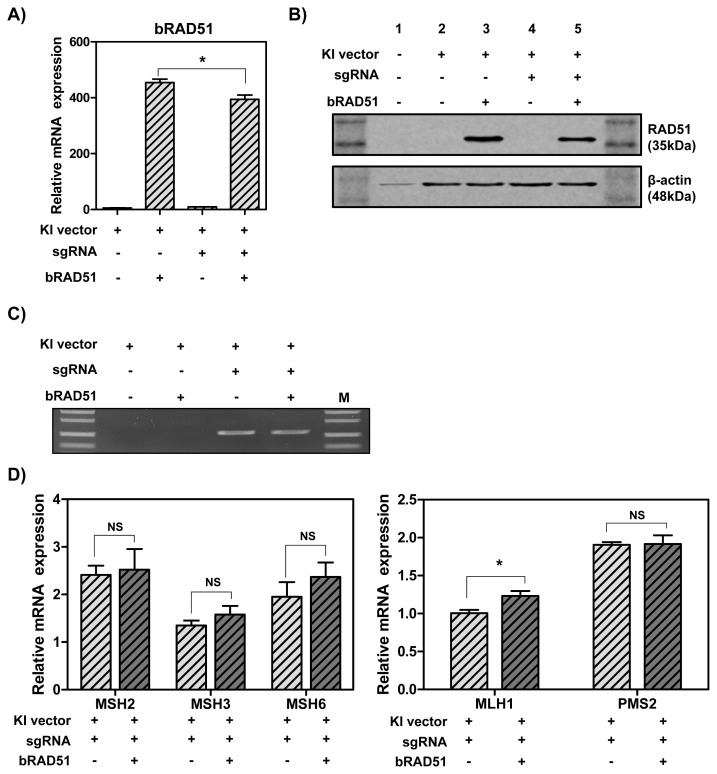
The expression levels of RAD51 and DNA MMR-related genes and CRISPR/Cas9-mediated knock-in efficiency in MAC-T cells induced overexpression of *RAD51* gene. (A) The mRNA levels of RAD51. (B) Protein expression of RAD51. (C) Polymerase chain reaction analysis of knock-in efficiency. M, DNA size marker (1 kb ladder). (D) The mRNA levels of DNA MMR-related genes. RAD51, RAD51 recombinase; MMR, mismatch repair; CRISPR/Cas9, clustered regularly interspaced short palindromic repeats/CRISPR-associated 9; MAC-T, bovine mammary epithelial cells. Error bars show the standard deviation from each sample. NS, no statistical difference; * p<0.05.

To determine whether overexpression of *RAD51* gene increases the knock-in efficiency by enhancing HR, MAC-T cells were transfected with hLF knock-in vector II, CRISPR-sgRNA expression vector and RAD51 overexpression vector. As a result, the mRNA (Figure 3A) and protein (Figure 3B) levels of RAD51 were significantly up-regulated. Also, the knock-in efficiency in the presence of CRISPR-sgRNA expression vector was higher than in the group without the expression vector. However, the overexpression of *RAD51* gene did not enhance knock-in efficiency (Figure 3C). Thus, to investigate whether DNA MMR-related genes affected *RAD51* gene, the mRNA levels of MMR-related genes were assessed by real-time qPCR. The results showed that MLH1 mRNA levels were slightly increased by RAD51 overexpression (1.23-fold), but other MutS and MutL homologs had no significant change (Figure 3D).

**Figure 4:**
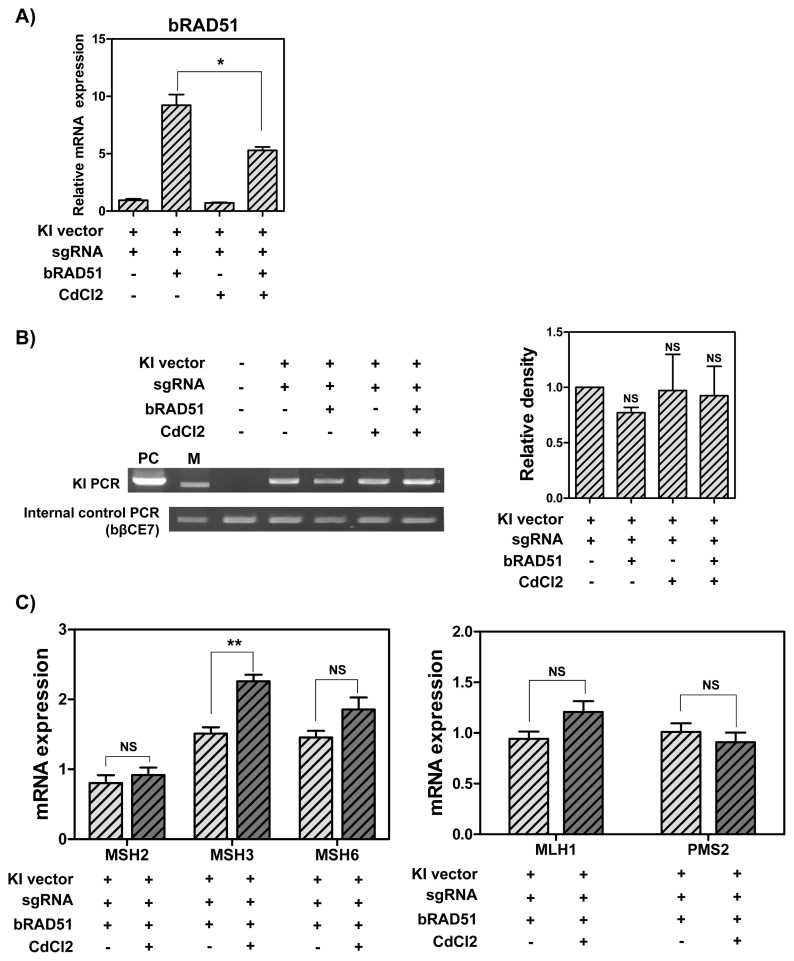
The mRNA levels of RAD51 and DNA MMR-related genes, and CRISPR/Cas9-mediated knock-in efficiency in MAC-T cells induced overexpression of RAD51 and treated with 1 μM CdCl_2_. (A) The mRNA levels of *RAD51* gene. (B) Polymerase chain reaction analysis of knock-in efficiency. M, DNA size marker (1 kb ladder). (C) The mRNA levels of DNA MMR-related genes. RAD51, RAD51 recombinase; MMR, mismatch repair; CRISPR/Cas9, clustered regularly interspaced short palindromic repeats/CRISPR-associated 9; MAC-T, bovine mammary epithelial cells. Error bars show the standard deviation from each sample. NS, no statistical difference; * p<0.05; ** p<0.01.

To determine whether RAD51 overexpression and down-regulation of DNA MMR-related genes increases the knock-in efficiency in MAC-T cells, cells overexpressing *RAD51* gene were treated with 1 μM CdCl_2_. As a result, RAD51 expression was decreased by CdCl_2_ treatment (Figure 4A). Also, the knock-in efficiency in the presence of CRISPR-sgRNA expression vector was higher than in the group without the expression vector. However, no significant difference was observed among the groups (Figure 4B). Next, the mRNA levels of DNA MMR-related genes were analyzed via RT-qPCR. As indicated in Figure 4C, increased MSH3 mRNA levels were observed (1.5-fold), but there was no significant difference in other DNA MMR-related genes in MAC-T cells overexpressing *RAD51* gene and treated with 1 μM CdCl_2_. All things considered, RAD51 overexpression and treatment with 1 μM CdCl_2_ did not increase the knock-in efficiency in MAC-T cells. Further, the mRNA levels of DNA MMR-related genes showed no significant difference.

### CRISPR/Cas9-mediated knock-in efficiency and expression levels of RAD51 and DNA MMR-related genes in MAC-T cells overexpressing CHK1

**Figure 5:**
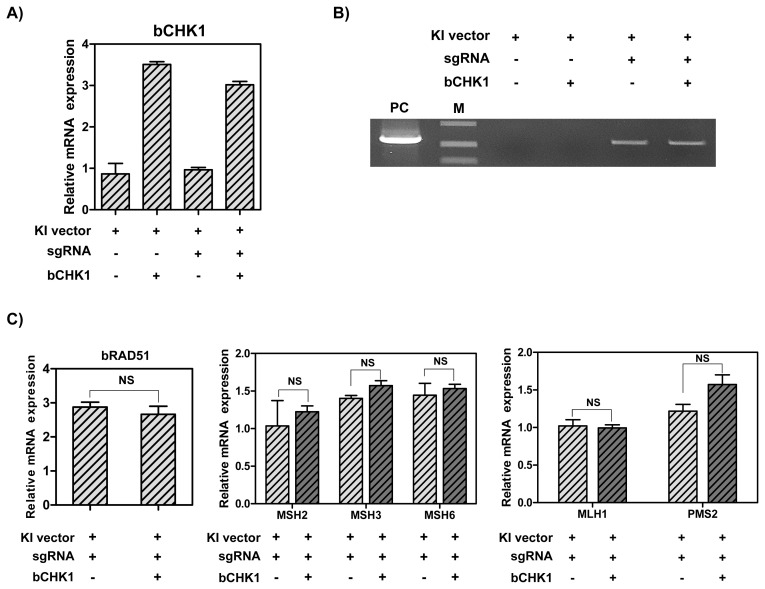
The mRNA levels of CHK1 and DNA MMR-related genes, and CRISPR/Cas9-mediated knock-in efficiency in MAC-T cells induced overexpression of *CHK1* gene. (A) The mRNA levels of *CHK1* gene. (B) Polymerase chain reaction analysis of knock-in efficiency. M, DNA size marker (1 kb ladder). (C) The mRNA levels of RAD51 and DNA MMR-related genes. CHK1, Checkpoint kinase 1; MMR, mismatch repair; MAC-T, bovine mammary epithelial cells; RAD51, RAD51 recombinase. Error bars show the standard deviation from each sample. NS, no statistical difference.

In order to determine whether the overexpression of *CHK1* gene can increase the knock-in efficiency by stimulating *RAD51* gene, MAC-T cells were transfected with hLF knock-in vector II, CRISPR-sgRNA expression vector and RAD51 overexpression vector. As a result, the CHK1 mRNA levels were up-regulated by CHK1 overexpression vector (3-fold) (Figure 5A). Also, the knock-in efficiency in the presence of CRISPR-sgRNA expression vector was higher than in the group without the expression vector. However, overexpression of *CHK1* gene did not increase the knock-in efficiency in MAC-T cells (Figure 5B). Next, the analysis of the mRNA levels of RAD51 and DNA MMR-related genes via RT-qPCR showed no significant change in RAD51 and DNA MMR-related genes following CHK1 overexpression (Figure 5C).

### CRISPR/Cas9-mediated knock-in efficiency and expression levels of RAD51 and DNA MMR-related genes in MAC-T cells overexpressing CHK2

**Figure 6:**
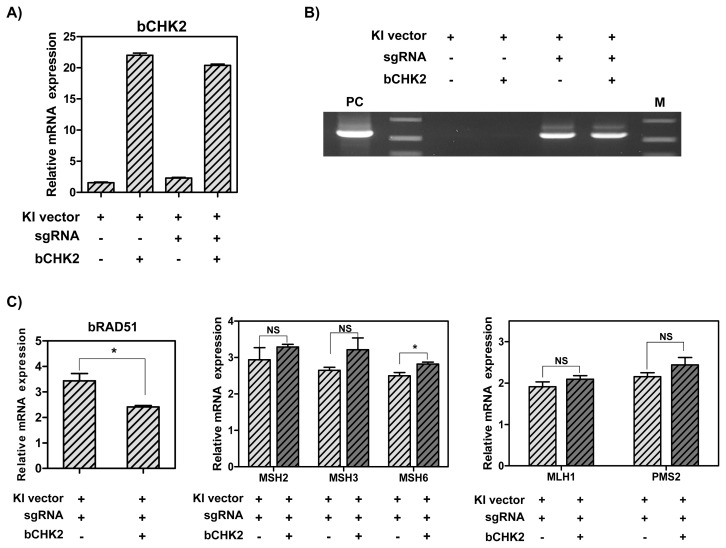
The mRNA levels of CHK2 and DNA MMR-related genes, and CRISPR/Cas9-mediated knock-in efficiency in MAC-T cells induced overexpression of *CHK2* gene. (A) The mRNA levels of *CHK2* gene. (B) Polymerase chain reaction analysis of knock-in efficiency. M, DNA size marker (1 kb ladder). (C) The mRNA levels of RAD51 and DNA MMR-related genes. CHK2, Checkpoint kinase 2; MMR, mismatch repair; MAC-T, bovine mammary epithelial cells; RAD51, RAD51 recombinase. Error bars show the standard deviation from each sample. NS, no statistical difference; * p<0.05.

To determine whether overexpression of *CHK2* gene increases HR-mediated knock-in efficiency in MAC-T cells by stimulating *RAD51* gene, the MAC-T cells were transfected with hLF knock-in vector II, CRISPR-sgRNA expression vector and CHK2 overexpression vector. First, it was observed that the mRNA levels of *CHK2* gene were up-regulated by CHK2 overexpression vector (20-fold) (Figure 6A). Also, the knock-in efficiency in the presence of CRISPR-sgRNA expression vector was higher than in the absence of the expression vector. However, the overexpression of *CHK2* gene did not increase the knock-in efficiency in MAC-T cells (Figure 6B). Next, the analysis of the mRNA levels of RAD51 and DNA MMR-related genes by RT-qPCR revealed a decrease in the mRNA levels of *RAD51* gene by 30% and a slight increase in MSH6 by 1.13-fold, but no significant difference was observed in other MutS and MutL homologs (Figure 6C).

## DISCUSSION

This study investigated whether the regulation of DNA DSB repair-related genes and MMR system was associated with knock-in efficiency of hLF knock-in vector in the β-casein gene locus of MAC-T cells.

As stated in the introduction, numerous studies have been demonstrated an increase in the HR-mediated knock-in efficiency. However, gene targeting via HR is strongly suppressed by DNA MMR-dependent anti-recombination when the DNA strands contain excessively mismatched nucleotides [16]. In general, metal compounds such as cadmium have been reported as mutagenic since they interrupt HR-related systems and cell cycle regulation [22]. Decrement of MMR activity after CdCl_2_ treatment was detected up to at least 5 μM in a dose-dependent manner [23]. This finding raises the question whether CdCl_2_ increased the knock-in efficiency by inhibiting MMR system.

In the present study, MAC-T cells were co-transfected with hLF knock-in vector II and CRISPR-sgRNA expression vector. Changes in knock-in efficiency and mRNA levels by CdCl_2_ treatment were also investigated. The PCR analysis revealed that CdCl_2_ treatment did not induce dose-dependent knock-in efficiency in MAC-T cells. This result does not support our hypothesis that knock-in efficiency is increased by CdCl_2_ treatment in a dose-dependent manner. However, based on the gradual decrease in mRNA levels of RAD51 and DNA MMR-related genes by increasing the treatment duration of CdCl_2_, our data support that CdCl_2_ treatment down-regulates the mRNA levels of RAD51 and DNA MMR-related genes depending on the duration of treatment [19,24]. Both MutSα and MutSβ complexes possess ATPase activity and initiate MMR by recognizing and binding to a mismatch. At this point, cadmium inhibits ATP hydrolysis of MutSα complex and as a result of this inhibition, binding to mismatched DNA by MSH2–MSH6 are reduced. Regarding the mechanism of down-regulation of MMR-related genes by cadmium, it is clear to say that cadmium can generate oxidative stress in cells and induce significant down-regulation of MutS complex composed of MSH2 and MSH6 [19]. Therefore, our results indicate that CdCl_2_ treatment does affect the mRNA levels of RAD51 and DNA MMR-related genes, and the mismatch recognition and excision functions of MMR system in bovine. However, the expression of MSH2 and RAD51 proteins was increased on day 1 and 2 by CdCl_2_ treatment, which was not consistent with the aforementioned results about mRNA expression levels. The binding sites of cadmium in RAD51 and DNA repair-related proteins have not been perfectly identified. Therefore, further studies are needed to investigate the detailed molecular mechanisms underlying DNA repair and inhibitory effects of cadmium.

In the present study, *RAD51* gene was overexpressed in MAC-T cells in order to assess whether RAD51 overexpression promoted knock-in efficiency. A recent study reported that the knock-in efficiency by CRISPR/Cas9-mediated HR was enhanced up to 2.5-fold by co-transfection of *RAD51* in brain neurons [25]. *RAD51* gene overexpression has not only revealed such positive results but also negative effects on initiation of HR. Overexpression of human RAD51 reduced DSB-induced HR in CHO cells and human cells [26]. In contrast to prior studies, our data indicated no significant difference in CRISPR/Cas9-mediated knock-in efficiency in MAC-T cells following RAD51 overexpression. It may reflect differences in levels of overexpression or host species of *RAD51* gene. Furthermore, the mRNA levels of MLH1 were slightly increased but other DNA MMR-related genes showed no detectable changes in MAC-T cells following RAD51 overexpression. These results suggest that *RAD51* gene overexpression may not substantially affect the mRNA levels of DNA MMR-related genes.

The limitation to recombination introduced by heterology can be overcome in MSH2-deficient cells [27]. Accordingly, it was hypothesized that CdCl_2_ treatment suppresses MMR activity in MAC-T cells overexpressing *RAD51* gene and thus increase HR-mediated knock-in efficiency. However, treatment of MAC-T cells overexpressing *RAD51* gene with 1 μM CdCl_2_ did not significantly affect the knock-in efficiency or mRNA levels of DNA MMR-related genes apart from *MSH3* gene, which was increased 1.5-fold. Cadmium affects the functions of eukaryotic MutSα complex (MSH2–MSH6) to bind to mismatched DNA via inhibition of ATP hydrolysis [28] but only at lethal concentrations [19]. In this study, we treated 1 μM CdCl_2_ to minimize unexpected mutations in cells and this concentration was able to down-regulate MMR-related genes but might be insufficient to block the binding of MutS complex to mismatched DNA, resulting in no significant change in knock-in efficiency. In addition, cadmium exposure to lower concentrations such as 1 μM stimulates DNA synthesis and cell proliferation in various cell lines, whereas further elevated concentrations are inhibitory. Furthermore, the reduction of MMR activity in a human cell extract by cadmium is concentration-dependent, but 1 μM was undetectable *in vitro* since MMR pathway was incomplete even in the absence of cadmium [23]. Our data correspond to this previous study and thus suggest the need to treat the cells with increased concentrations of CdCl_2_ to inhibit MMR activity.

Furthermore, disruption of CHK1 expression inhibited the formation of RAD51 foci and resulted in high levels of unrepaired DSB; however, DNA damage was repaired efficiently in CHK1-proficient cells. The MMR system at the DNA damage sites facilitates CHK2 phosphorylation by ATM and enables MSH2 binding to CHK2 [29]. In this respect, it was supposed that CHK1 or CHK2 overexpression increases the knock-in efficiency in MAC-T cells by stimulating RAD51 localization and enhancing HR frequency. Based on our results, no significant differences were found in knock-in efficiency and mRNA levels of RAD51 and DNA MMR-related genes in MAC-T cells following CHK1 overexpression. Accordingly, these results may be explained by low levels of CHK1 overexpression. Similarly, no significant difference in CHK1 overexpression was detected. It is plausible that CHK1 and CHK2 kinases do not critically stimulate RAD51 and HR. At this point, the functions of the two kinases, CHK1 and CHK2, remain to be elucidated in response to DNA double-strand breaks triggered by CRISPR/Cas9 system.

In summary, the inhibition of the mRNA levels of RAD51 and DNA MMR-related genes in MAC-T cells was triggered by CdCl_2_ treatment. However, no detectable differences in knock-in events were confirmed in MAC-T cells treated with CdCl_2_ or overexpressing DNA DSB repair-related genes. Further studies in relation to the DNA DSB repair-related genes apart from CHK1, CHK2, and DNA MMR-related genes are needed to effectively regulate HR. Besides, additional interaction between DNA DSB repair-related genes and DNA MMR-related genes need to be revealed. Presumably, the absence of significant changes in knock-in efficiency in MAC-T cells is attributed to the distance between the sites of modification and cleavage by the CRISPR-sgRNA system in the knock-in vector. According to the study in 2014, introduction of the cleavage site in close proximity to the altered locus in the design of sgRNAs for HR, has a strong effect on the knock-in efficiency [30]. However, the knock-in vector used in this study was designed to carry a 5′ homology arm, which is distant from the DSB point. Further, differences in sensitivity to cadmium and functions of MMR system in mammalian cell types and species suggest the need for further studies in other types of mammalian cells, such as mouse or human.

## Figures and Tables

**Table 1:** Primers used for quantitative real-time polymerase chain reaction analysis of specific genes

Gene	Primer name	Sequence (5′→ 3′)	Size (bp)
*Rplp0*	mRplp0 F	CGACCTGGAAGTCCAACTA	109
	mRplp0 R	ATCTGCTGCATCTGCTTG	
*MSH2*	bMSH2 CDS S	ATGAGGAAGCCCAGAATGCC	85
	bMSH2 CDS AS	ACATCGTTGAGTGTCTGCATC	
*MSH3*	bMSH3 CDS S	TATCAGGACAGGAGTTTATGATT	81
	bMSH3 CDS AS	TGCTTCCAATCTTAACCCAATC	
*MSH6*	bMSH6 CDS S	TGCTTGTGGATGAATTAGGAAG	88
	bMSH6 CDS AS	ATGTTCTCAGCAAGTTCTTTCA	
*MLH1*	bMLH1 CDS S	TTATCGGAGCCAGCACCAC	96
	bMLH1 CDS AS	AAGTCCTTCCTTGGGACCAT	
*PMS2*	bPMS2 CDS S	AGGCTTATAGCACCTCAGACT	94
	bPMS2 CDS AS	AGTCAAAGCCATTCTTCCTGAA	
*RAD51*	bRAD51 CDS S	TTTCAGCCAGGCAGATGCAC	79
	bRAD51 CDS AS	ACCACTGCTACACCAAACTCA	
*CHK1*	bCHK1 CDS S	TCCCAGCCTACTTGTCCTGA	100
	bCHK1 CDS AS	TTTGACTAAGCGCTGCCAGG	
*CHK2*	bCHK2 CDS S	AGAGAAGGCTCTGGATCTCG	94
	bCHK2 CDS AS	CTGAAGCCACGGGTGTCTT	
